# Mitonuclear conflict and cooperation govern the integration of genotypes, phenotypes and environments

**DOI:** 10.1098/rstb.2019.0188

**Published:** 2019-12-02

**Authors:** David M. Rand, Jim A. Mossman

**Affiliations:** Department of Ecology and Evolutionary Biology, Brown University, 80 Waterman Street, Box G, Providence, RI, USA

**Keywords:** mitonuclear, epistasis, conflict, cooperation, GxG, GxE

## Abstract

The mitonuclear genome is the most successful co-evolved mutualism in the history of life on Earth. The cross-talk between the mitochondrial and nuclear genomes has been shaped by conflict and cooperation for more than 1.5 billion years, yet this system has adapted to countless genomic reorganizations by each partner, and done so under changing environments that have placed dramatic biochemical and physiological pressures on evolving lineages. From putative anaerobic origins, mitochondria emerged as the defining aerobic organelle. During this transition, the two genomes resolved rules for sex determination and transmission that made uniparental inheritance the dominant, but not a universal pattern. Mitochondria are much more than energy-producing organelles and play crucial roles in nutrient and stress signalling that can alter how nuclear genes are expressed as phenotypes. All of these interactions are examples of genotype-by-environment (GxE) interactions, gene-by-gene (GxG) interactions (epistasis) or more generally context-dependent effects on the link between genotype and phenotype. We provide evidence from our own studies in *Drosophila*, and from those of other systems, that mitonuclear interactions—either conflicting or cooperative—are common features of GxE and GxG. We argue that mitonuclear interactions are an important model for how to better understand the pervasive context-dependent effects underlying the architecture of complex phenotypes. Future research in this area should focus on the quantitative genetic concept of effect size to place mitochondrial links to phenotype in a proper context.

This article is part of the theme issue ‘Linking the mitochondrial genotype to phenotype: a complex endeavour’.

A given gene will manifest itself in different ways depending on the complex of other genes surrounding it. For this gene, that complex or genotype will comprise its genotypic environment, in which it expresses itself

—Sergei Chetverikov, 1926 [1, p. 223]

## 1. Introduction

Mitochondria influence most phenotypes in all eukaryotes. This may be true for other subcellular structures like ribosomes, the endoplasmic reticulum or centrioles, but mitochondria are so much more interesting because they have their own polyploid genome that is distributed across the cytoplasm and is distinct from the nuclear genome. This dual genomic architecture of eukaryotes is an exquisite example of evolutionary contingency [2] and arguably the most successful co-evolved mutualism in the history of life [3]. The union of an archaebacterium and a eubacterium that has been resolved as a eukaryotic cell consolidated distinct genetic, biochemical and ecological systems into a novel life form that permitted an explosion of biodiversity [4]. The origin and evolution of mitochondria have been credited as causal factors in the evolution of eukaryotes, multicellularity, the origin and maintenance of sex, the predominance of two mating types, the driving force behind speciation, the cause of ageing, the source of male fitness variation, among others. These ideas were clearly articulated almost 30 years ago by Cosmides & Tooby [5], which have been reviewed extensively [6–10] and are revisited by papers in this issue. Cosmides & Tooby [5] were the first to place this broad sweep of ideas in the context of intragenomic conflicts, and these ideas have been extended more recently [9,11,12]. Like any merger and acquisition, the interest of each unit differs, which is the source of the conflict. But some form of cooperation must ensue for the new emergent unit to be successful [13].

Here we will leave the macroevolutionay aspects of this topic to others and focus on the microevolutionary processes by which mitochondria are part of the link between genotype and phenotype. We revisit the concepts of mitonuclear conflict and cooperation from the perspective of gene-by-gene (GxG) and genotype-by-environment (GxE) interactions with a focus on experimental, quantitative genetic approaches in current day organisms. We propose the most important role that mitochondria play in linking genotype to phenotype is through their context-dependent epistatic and GxE interactions with the nucleus and the environment. These interactions are the basis of putative conflicts and cooperation that govern ongoing mitonuclear evolution and are the legacy of macroevolutionary conflict resolution. Understanding the link between genotype and phenotype is a central challenge of biomedical research, and while mitochondria are one of many players in this link, the mitonuclear interactions are the integrative context for discovering the causal mechanisms in the genotype–phenotype map.

## 2. Conflict, cooperation and the quantitative genetics of mitonuclear interactions

Because mitochondria are central to cellular metabolism, and many examples of mitochondrial DNA (mtDNA) mutations causing human disease have been documented, there is no doubt that mitochondria play some role in the genotype–phenotype map. How mitochondria may be driving evolutionary changes in the link between genotype and phenotype, and whether conflict and cooperation are part of this dynamic needs to be addressed with experimental analyses. The most interesting and tractable contexts for these experiments involve the conflicts between mtDNA and nuclear genotypes, between mitonuclear genotypes in different sexes and between mitonuclear genotypes in different environments. To demonstrate conflict, one must show that the phenotype of one of these factors is compromised (or not optimized) by the presence of the other factor. To demonstrate cooperation, one must show that interaction between the factors allows one or more of the factors to enjoy an improved phenotype, or longer persistence in populations, owing to the fitness of the emergent higher unit [13–15]. This, in turn, requires functional polymorphism among mtDNAs and interacting nuclear genes.

### (a) Nuclear genes in mitochondrial environments

**Figure 1.**
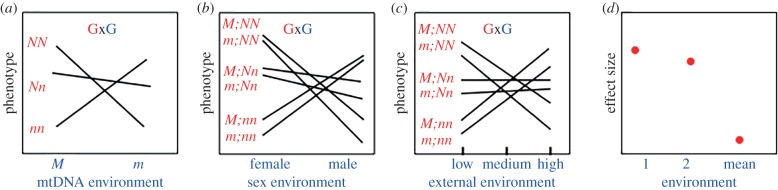
Mitochondrial effects are dominated by epistatic and environmental interactions. (*a*) illustrates a mitonuclear epistatic (GxG) interaction for a phenotype. The three nuclear genotypes at an autosomal locus (*NN*, *Nn*, *nn*) might show different norms of reaction across alternative mtDNA backgrounds (*M* and *m*). (*b*) Different mitonuclear genotypes (denoted as mtDNA; nuclear genotype) may have different phenotypes in different sexes. Here, the sex of the organisms provides a distinct environment for the genotype. (*c*) A typical genotype-by-environment (GxE) interaction where alternative genotypes have different phenotypes across a range of environments. The relative contributions of mtDNA and nuclear genes to these phenotypes are context dependent. Importantly, (*d*) demonstrates that the effect size for a given mtDNA or genotype might be large in any one environment but may be very small when averaged across all genetic and environmental backgrounds encountered in nature (i.e. marginal effect sizes are small). (Online version in colour.)

The opportunity for mtDNA and nuclear factors (or any genetic factor) to influence phenotype depends on the quantitative genetic concept of the *effect size* or the standardized difference in mean phenotype between individuals of two allelic or genotypic classes. For a diploid nuclear locus, the homozygous effect, *a*, would be half the difference in mean phenotype between alternative homozygotes (e.g. for the phenotypes of *NN* versus *nn* homozygotes, *a* = (*NN* − *nn*)/2). This is typically quantified as the proportion of the phenotypic standard deviation in the mapping population (*σ_P_*): thus, effect size = *a*/*σ_P_* [16]. For haploid mtDNAs, this would be the difference in mean phenotype between individuals carrying alternative mtDNAs (e.g. for the phenotypes of *M* versus *m* mtDNA haplotypes, *a* = *M*−*m* and effect size = *a*/*σ_P_*). In natural populations, the strength of an effect size would be the mean of each effect size across all the genetic backgrounds that a particular nuclear or mtDNA allele finds itself. Because functional polymorphism is well known in both nuclear and mtDNA genomes in nature, conflicts will arise if there are nuclear backgrounds in which either the *M* or the *m* mtDNA is not fit; and likewise for the *N* or the *n* nuclear allele in either the *M* or *n* mtDNA background. In short, each genetic factor provides an environment for the other (figure 1). Theory and experimental work demonstrate that for haploid mtDNA, this conflict will be resolved with the elimination of one mtDNA variant due to non-zero effect sizes for mtDNA that cannot be easily maintained in haploid systems even with autosomal modifiers [17–19]. However, when interacting nuclear loci are X-linked (in XX/XY systems), the dynamics of the nuclear variation can help maintain joint nuclear and mtDNA variation in a system that is consistent with cooperation [3]. The long-term fate of these polymorphisms depends on whether the mitonuclear epistatic interactions are positive or negative, which in turn will determine whether conflicts are resolved through cooperation or purging of polymorphisms. In general terms, the effect sizes and signs of the mtDNA, nuclear and interaction effects determine the dynamics of the system, and these factors probably vary in unpredictable ways in nature. A more complete examination of these interactions has been presented by Wade & Drown [20].

### (b) Mitochondrial genes in sex environments

The uniparental transmission of mtDNA presents a clear context for different links between mtDNA genotype and phenotype, especially with respect to sex-specific effects. Maternal transmission of mtDNA and biparental transmission of nuclear chromosomes establishes conflict, where mtDNA alleles' interests are linked to female fitness, and nuclear alleles’ interests depend on both male and female fitness [5,21]. This transmission dynamic allows selection to operate on mtDNAs in females, but not in males. Haploid selection should purge female-limited mtDNA effects but allow male-limited deleterious mtDNA effects to persist in populations if they were neutral or beneficial in females. It follows that mtDNA variance in effect sizes should be small in females, but large in males, a condition that has been dubbed Mother's Curse [22]. Evidence in support of this has been provided in *Drosophila* [3,23,24] and humans [25], but it is not a universal phenomenon [26–29].

A key question in Mother's Curse is whether nuclear modifiers of male deleterious mtDNA effects can arise. It has been shown that inbreeding or assortative mating can minimize the impact of Mother's Curse [30,31] by permitting nuclear modifiers to be effective despite a lack of mtDNA transmission in males. As noted above, the dynamics of mtDNA–X-chromosome interactions can permit cooperative interactions that might compensate for male deleterious effects [3]. The Y chromosome provides one source of Mother's Curse modifiers given its male-limited transmission, which should facilitate compensatory mutations [32]. And in ZW species where females are the heterogametic sex, the co-transmission of genetic elements provides a different set of conflicts and cooperative interactions that can modify Mother's Curse and introduce a parallel Father's Curse [32]. A critical question in these studies concerns the generality of the Mother's Curse phenomenon, given the studies that do and do not support the predicted pattern [24,26]. It seems reasonable that the context-specific nature of mitonuclear epistatic interactions could enhance or mask Mother's Curse patterns owing to either positive or negative fitness trajectories for joint mitonuclear genotypes [20]. Connallon *et al*. [33] examined the population genetics parameters that drive the Mother's Curse phenomenon and found that large and small effective population sizes may promote it, while intermediate effective population sizes may not result in this pattern. The significance of this depends again on the effect size of the mtDNA and nuclear background alleles, averaged across their respective genetic backgrounds and, most notably, across each sex background. In this way, we consider sex as yet another component of the genetic background or a form of environmental background.

### (c) Mitonuclear genotypes in external environments

Virtually all phenotypes have some degree of environmental sensitivity, also known as phenotypic plasticity. For mitochondrial and nuclear genetic interactions, their central role in cellular function should be highly responsive to environmental variation. Here an analogous form of conflict and cooperation presents itself, and again the critical factor is the effect size of each mtDNA or nuclear allele across the range of environments that either may encounter in the realization of an organism's life history. An allele that is favoured in one environment but deleterious in another—a condition of crossing reaction norms or GxE interactions—could be maintained in the entire population. This describes the Levene model of a balanced polymorphism [34], and the environment generates a form of conflict for the genetic polymorphism. The conflict can be resolved or purged, depending on the genetic exchange between niches or relative proportions of alternative niches in the overall environment. Alleles or mtDNAs showing GxE could have very large effect sizes in the extremes of the environmental range but small effect sizes averaged across all environments (figure 1). This GxE condition is exactly analogous to the GxG interaction for a mitonuclear epistasis that might maintain, or at very least, influence genetic variation.

### (d) Mitochondrial metabolism as a quantitative genetic integrator

It is our opinion that experimental studies demonstrating mtDNA phenotype effects in one or two nuclear backgrounds or environmental contexts by no means establish that this pattern is general in the natural world. This is an old and thorny problem in the experimental population and ecological genetics. The basis of this opinion lies in the most general aspect of cellular metabolism: it has evolved varied pathways to provide functional plasticity to respond to varied environmental stresses. Mitonuclear interactions provide the mechanistic basis for common features of epistatic GxG and GxE interactions [35]. Mitochondria are now recognized as much more than ATP factories, with central roles in nutrient and redox sensing, as well as diverse cellular signalling pathways [36,37]. As such, mitochondria are central integrators of the external and internal conditions that can translate biochemical reagents into phenotypic traits. This role as an integrator of cellular function is probably a legacy of the cycles of conflict and cooperation that resulted in the emergence of mitochondria from the original endosymbiosis event [9]. This transition involved the consolidation of distinct biochemistries where waste products from the eubacterium may have provided nutrients for the archaebacterium and vice versa [38]. The metabolites that were the currency for these early interactions have been retained as part of the complex set of signals that maintain mitonuclear communication today. Examples include electron coupling reactions of the respiratory chain involving H+ and NAD+/NADH balance [39], calcium signalling [40,41] and ADP/ATP transport [9,42,43], among many others [44].

An important component of these signalling processes is a distinct role for the environment in the nature of the signal: most of these metabolites have different concentrations inside and outside of cells, and their function provides a critical signalling link between the external ecological and the internal genetic environments. The function of mitochondria depends critically on the integration of 37 mtDNA-encoded gene products and approximately 1200 nuclear-encoded gene products, which is mediated by these diffusible and transported metabolites that serve as messengers in the anterograde and retrograde signals between the two genomes. With signalling mediated by molecules that originate both outside the organism (nutrients, oxygen) and inside mitochondria (‘mitobolites’, [36]), the distinction between G×E interactions and G×G interactions becomes blurred. Moreover, the reality of these biochemical and ecological interactions directs the focus on the question of mitochondrial genotypes' effects on phenotype, to the broader context of mitonuclear interactions’ effects on phenotype [35]. This perspective motivates a quantitative genetic approach to mitonuclear interactions: mitochondrial functions as quantitative traits.

The central challenge of quantitative genetics is to define the genetic architecture of phenotypes: what genes are involved, how big are their effects, do they interact with each other and with biotic and abiotic environmental variables. To date, an unprecedented number of studies have looked at the effects of individual single-nucleotide polymorphisms (SNPs) on phenotypic variation or disease risk in a genome-wide association (GWA) framework. The magnitude of this scientific endeavour in humans alone is over 3764 publications and 107 785 SNP-trait associations as of January 2019 (https://www.ebi.ac.uk/gwas/). However, GWA methods have produced two unexpected results [45]: the most significant genes in these studies account for only a small fraction of the total genetic variation for traits, and many of the GWA hits are in non-coding regions of the genome. The first result, known as the ‘missing heritability’ problem [46], implies that many genes of small effect, or gene interactions, that contribute to trait variation remain undetected. The second result implies that gene regulation is an important component of trait variation. These results have motivated new models of the genetic bases of phenotypic variation. The simple polygenic model invoking a few strong-effect genes with several modifier loci is now incomplete. The GWA data for height [47] are consistent with the classical infinitesimal model of R.A. Fisher [48], and the role of non-coding gene-regulatory SNPs in complex traits invokes gene interaction in phenotypic variation. Boyle *et al*. [45] interpret these findings as an ‘omnigenic’ model in which ‘core’ genes at the hubs of regulatory networks are connected through numerous ‘peripheral’ genes that modify core, cellular functions. Because peripheral genes outnumber core genes 100-fold, the omnigenic model seeks to integrate the puzzle of missing heritability with the phenotypic effects of non-coding SNPs. While this view extends the infinitesimal model to an extreme, interactions between core and peripheral genes are implicit components of the omnigenic model.

How does this perspective connect with the role of mitochondria in the phenotype–genotype map? It has been noted that these GWA studies neglect to include SNPs in mtDNA [49] and often ignore the entire X-chromosome. We argue that mitonuclear genetic variation embodies highly complex and pervasive GxG and GxE interactions that modify organismal fitness and function in non-additive ways and is an ideal context for evaluating the nature of the polygenic and omnigenic models. This view has been informed by empirical studies in the fruit fly *Drosophila melanogaster* and other model organisms and follows from the enormity of functions in eukaryotic cells that are governed by mitonuclear cross-talk [50,51]. We suggest that the mitochondrion is a major source of pleiotropic and epistatic phenotypic effects but urge the field to avoid singular interpretations of mtDNA as the primary driver of simplistic models of fitness and adaptation. The quantitative genetic reality of average effect sizes and a systems biology approach of integration and interaction are warranted to understand how evolutionarily conserved genetic pathways interact with the environment to modify phenotypes and fitness. We recognize that mtDNA is inherited as a single linkage group encoding genes for multiple proteins of the core energy-producing function in cells, so it can be considered a sort of super gene complex that may have a disproportionately large effect on a per-base-pair basis. But the impact of mtDNA to phenotypic variation is often a fraction of the nuclear or environmental effects in those cases where the main effects of mtDNA, nuclear DNA and environmental factors have been quantified. [3,19,26,52,53]. Often, the interaction effects with mtDNA are greater than the main effect of mtDNA, so its effect on fitness and adaptation may be mediated by epistatic rather than main effects. To claim that mtDNA is more important than the entire nuclear genome as a driver of organismal fitness and adaptation does not seem warranted by these data.

## 3. Nonlinear interactions: complications for phenotypic prediction

For over a century, it has been known that genes rarely operate in isolation [54–57], and GxG nonlinear interactions (epistases) are widespread phenomena [58,59] underlying numerous phenotypic traits [60,61]. On first principles, higher-order epistatic interactions are challenging to describe because the allelic variation at individual interacting loci may demonstrate zero or negligible statistical trait associations [62,63]. Instead, the effects of alleles can be dependent on the context of other alleles they find themselves interacting with [57], resulting in ‘cryptic’ genetic and phenotypic variation [64]. The qualifying term ‘cryptic’ is used to describe unforeseen or unpredictable outcomes of allelic pairing (or three, four or *n*-level higher-order interactions) that are revealed under very specific genetic contexts. As a result, predicting the origin, identity and effects of nonlinear and multiplicative genetic interactions is the central challenge for quantitative and medical genetics.

Despite these complications, large effect size interactions between loci have been successfully mapped in polygenic human disease (ankylosing spondylitis: HLA-B27 and *ERAP1* [65]; psoriasis: *ERAP1* and HLA-Cw6 [66]). Numerous genetic models are being developed to detect genome-wide genetic interactions underlying complex human traits [67–69]. Performing exhaustive searches for pairwise (and higher-order) genome-wide interactions is a computationally intensive process and alternative data mining and machine learning approaches are now routinely used to accelerate statistical analyses of epistatic genes [68]. The outcomes of these genome-wide screens produce population-level associations usually involving thousands of individuals. However, the more relevant granularity for personalized genomics or medicine is at the individual level. This presents a problem since even genetically identical individuals (e.g. identical twins) demonstrate variable susceptibility (penetrance) to common disease [70–72]. For these reasons, it has been suggested that ‘we may never be able to make accurate predictions about disease risk in individuals using genetics alone’ [73, p. 168].

Given the phenotypic heterogeneity of genetically identical individuals, the role of environment and epigenetics has been strongly implied in complex traits. For example, in isogenic *Drosophila* strains, there is heritable intragenotype phenotypic variability [74], even when tested in controlled environments. Likewise, random variability in quantitative biological traits is routinely observed in inbred rodent strains in highly controlled environments with standardized husbandry [75]. Furthermore, GxE interactions have been shown to vary in isogenic *Caenorhabditis elegans* [76]. The complexity of GxG, GxE and potentially higher-order effects requires that the genotype–phenotype landscape changes in different high-dimensionality environments [77]. To fully dissect, these higher-order effects will require extensive study in model organisms in controlled abiotic (and genetic [35]) environments. Even after extensive study, it may not be possible to determine if genetic inference from inbred or isogenic model organisms can translate to large effect sizes in the context of outbred, heterozygous humans. It is likely that the genetic effect sizes required for robust inference of mtDNA may be generally very low, and sample sizes for good support, prohibitively large in the face of environmental variability [78].

## 4. Experimental approaches to map mitochondrial genotypes to phenotypes

**Figure 2.**
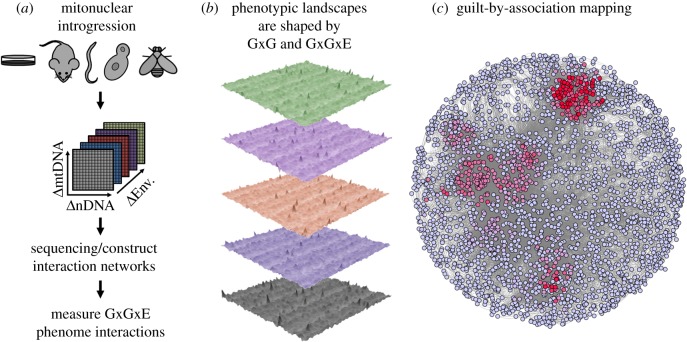
Mitonuclear phenotypic landscapes. (*a*) A number of genetically tractable model organisms should be introgressed to test the phenotypic effects of three axes of variation: (i) nuclear DNA (ΔnDNA), (ii) mitochondrial DNA (ΔmtDNA) and (iii) environment (ΔEnv.). A combination of genome DNA sequencing, RNA-seq(uencing) and whole-organism phenome measurements can help construct a comprehensive map of the phenotypic landscape. (*b*) shows five simulated datasets of 6400 mitonuclear genotypes (80 mtDNAs × 80 nDNAs), responding randomly to diet (alternative colours). Regions of these landscapes that are modified by environment (*b*) are associated with GxGxE loci. Comprehensive sequencing of these lines can help identify associated modifying (or sensitive) loci. Likewise, gene–gene or protein–protein interaction networks (*c*) with overlapping across-taxa hotspots of mitonuclear dysregulation can pinpoint mtDNA-sensitive genes or sub-networks. This is ‘guilt-by-association’ across evolutionarily divergent species, to map evolutionary conserved networks and pathways [73]. These identified regions (red hotspots in (*c*)) can then be genetically manipulated to narrow down the search space for mitonuclear epistasis and/or GxGxE for forward genetics confirmation. Shown is a subsection of the *Drosophila* protein–protein interaction network [79]. (Online version in colour.)

As mtDNA is maternally inherited and non-recombining in most animal species, genetic crossing designs in model organisms allow the precise placement of mtDNAs with alternative nDNA backgrounds. By introgressing distinct mtDNA haplotypes onto isogenic nuclear chromosomal backgrounds (nDNAs), a factorial panel of mitonuclear variation can be assayed to identify the contribution of nDNA, mtDNA and their epistatic interaction on fitness-related traits (figure 2). The elegance of this approach is, however, moderately restricted to recessive mutations with large effect sizes and few genetic and environmental interactions. The roles of dominance and heterozygosity on mtDNA interactions remain poorly understood. To date mitonuclear effects have been tested by generating appropriate crosses and assaying genotypes across model animal species: *Drosophila* [23,24,27,28,80–84], nematodes [85], marine copepods [86–89], yeast [90–92], rodents [93,94], wasps [95]; human and rodent cybrid cell lines [96–100] and plants [101].

Epistasis is widespread in *Drosophila* [102] and complex life-history traits are dominated by a largely polygenic architecture. This provides an opportunity to test predictions of the omnigenic model as well as uncovering the common pathways that are influenced by mtDNA and that underlie complex traits and disease. Using *Drosophila*, we take a forward genetics approach to understand epistasis and exploit the known physical interactions between mtDNA and nDNA gene products that should coevolve in lineages [7]. We genetically perturb this co-evolved system by introgressing mtDNAs from within- and between-species of *Drosophila* onto various nuclear genetic backgrounds to quantify phenotypic effects from the whole organism to cellular respiration levels of the phenome. The potential GxG interaction space across all genes in the nuclear and mitochondrial genomes is excessively large, and by restricting our genetic model to the approximately 1200 nuclear genes and 37 mtDNA genes, we effectively narrow this search space to a more manageable and interpretable genotypic environment (figure 2). Ultimately, the goal is to understand how phenotypes vary across the GxG interaction (and fitness [103]) landscape to then make accurate predictions of the phenotype from known nDNA genotypes, sequences or mtDNA haplotype (‘whole-genome reverse genetics' [73,104]).

### (a) Lessons from mitonuclear fruit flies

Three important lessons have emerged from our mitonuclear introgression analyses in *Drosophila*. First is the falsification of a simple mitonuclear coadaptation model. A simple coadaptation model would predict that coevolving entities would function better when associated than when either entity is transplanted on to a foreign host or associate with which it has not co-evolved. We expect mtDNA from a different species to disrupt co-adapted mitonuclear interactions when placed on the nuclear background of a species with which it has not co-evolved [7]. In multiple instances, we have found that *D. melanogaster* flies carrying a *Drosophila simulans* mtDNA that diverged approximately 2 Ma and which differs at approximately 100 amino acid positions across the mtDNA genes encoding subunits of the electron transport chain, and approximately 400 additional substitutions in synonymous sites, transfer RNAs (tRNAs) and ribosomal RNAs (rRNAs), have very subtle phenotypic effects [105]. We also note that *D. melanogaster* flies carrying a *Drosophila yakuba* mtDNA that diverged approximately 10 Ma, and which differs at approximately 1018 nucleotide positions including approximately 171 amino acid changes across the protein-coding genes does not alter longevity [106] or climbing ability (A. Spierer and D. Rand 2015, unpublished data). This means that extensive mtDNA divergence has resulted in an effect size not different from zero and that all fixed differences are functionally neutral, or that a complex history of epistatic, compensatory or cooperative mutations has left no profound signal of a phenotypic effect for these *Drosophila* species pairs. A similar result has been found in mitonuclear cybrid cell lines in mice: cytochrome *c* oxidase (COX) activity is not disrupted in inter-species transplants among mice species, but in rat-mouse cybrids, a breakdown in function is observed [107]. One possible explanation for this is that purifying selection on mtDNA mutations has removed the strong-effect mutations allowing functionally neutral mutations to fix between species, which is consistent with many studies in molecular evolution [108,109].

**Figure 3.**
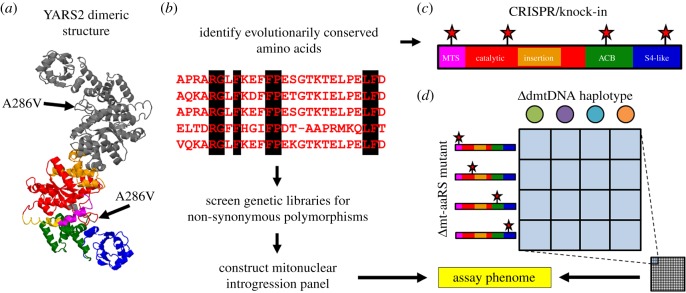
‘Candidate’ mitonuclear epistasis mapping. The YARS2 gene (the human homologue of *Drosophila Aatm*) is shown in its dimeric crystal form in (*a*). We have highlighted the causative amino acid polymorphism associated with a large-effect negative mitonuclear epistasis in *Drosophila*. The SNP is highly conserved across metazoa yet mutated in the negative epistatic *OregonR* nuclear background. The *tRNA^Tyr^* molecule docks the YARS2 protein and is presumably modified by the polymorphic locus. Different coloured regions represent different protein domains (labelled in (*c*)). Given this knowledge, mitochondrial aminoacyl-tRNA synthetases (mt-aaRSs) may be rewarding proteins to use for targeted (candidate) perturbation. There are many ways to conduct this, and we show two methods. (*b*) First, highly conserved mt-aaRS gene sequences across taxa can be screened in genetic panels of fully sequenced nuclear backgrounds. Any genetic lines with non-synonymous amino acid polymorphisms at conserved loci would be good candidates to introgress with a panel of mtDNA haplotypes demonstrating variation in the cognate mtDNA-encoded tRNA gene. (*c*) Alternatively, targeted knock-in can be used at any regions of the gene of interest, e.g. YARS2 (shown in (*a*,*c*)). To accomplish this, *in silico* knock-ins could be made to select the highest likelihood negative perturbation [114]. Red stars in (*c*) show example targets for mutation via CRISPR. In figure 3*d*, the mitonuclear panel of genotypes is represented by different mtDNA haplotypes (columns) and nuclear backgrounds with specific mt-aaRS polymorphisms (rows). The panel can be scaled-up to include as many mt-aaRSs and mtDNA haplotypes as required. These constructs may produce lethal offspring, but both approaches would identify mitonuclear interacting loci with effects on the phenome. MTS: mitochondrial targeted sequence (pink), catalytic: catalytic domain (red), insertion: insertion domain (orange), ACB: alpha-helical domain (green), S4-like: anticodon binding domain (blue). (Online version in colour.)

Second, and related to the first, is that the phenotypic effects of mtDNA mutations are greater within species than between species: alternative *D. melanogaster* mtDNAs have measurable effects on phenotype, as do alternative *D. simulans* mtDNAs on *D. melanogaster* nuclear backgrounds, but the interspecific effects are weak [26,105]. This demonstrates the importance of using an mtDNA outgroup in experimental introgression studies, as a linear relationship between mtDNA divergence and phenotypic effect is a simple predictive model of mitonuclear coevolution. The third lesson is that the effects of each mtDNA depend on the nuclear background and the environmental conditions. This latter point is true even for a mitonuclear introgression genotype that mimics a strong mitochondrial disease [80,105,110]. The *w501* mtDNA haplotype from *D. simulans* paired with the nuclear chromosomes from *D. melanogaster OregonR*—a common laboratory strain—has a suite of developmental, biochemical and fecundity defects. The causative loci in this are a non-synonymous SNP in the nuclear-encoded mitochondrial tyrosyl tRNA synthetase gene (*Aatm*) and a polymorphism in the anticodon stem of its cognate mtDNA-encoded *tRNA^Tyr^*. The exaggerated deleterious phenotype is the first example of mitonuclear epistasis mapped to single-nucleotide levels in *Drosophila* [80,105]. Despite its strong epistatic effect size, this mtDNA mutation has only mild effects on a different nuclear background. Moreover, the epistatic ‘disease’ condition is dramatically reduced at low temperatures, indicating mitonuclear GxE, or GxGxE effects [111,112]. This is not a peculiar consequence of a *D. simulans* mtDNA in a *D. melanogaster* nuclear background, because five other *D. simulans* mtDNAs on those same nuclear backgrounds show no evidence for the epistatic effects. Crucially, it is the chance encounter of mtDNA and nuclear alleles that confers the deleterious phenotype, but each polymorphism was segregating in its own populations with minimal phenotypic effects. While tRNAs represent only about 10% of the coding sequence of mtDNA, these genes represent about 40% of the known cases of mitochondrial disease. This points to a critical role for tRNA charging and protein synthesis in disease conditions, and probably in many mitochondrial phenotypes [113]. The biology of mt-tRNA interactions with their cognate nuclear-encoded tRNA synthetases provides a compelling model for the mechanistic dissection of mitonuclear interactions (figure 3).

## 5. Phenotypic repeatability is crucial

Motivated by the frequency of epistatic interactions in our initial mtDNA × nDNA panel, we built a larger panel of 72 mitonuclear genotypes [26] based on fully sequenced nuclear genomes from the *Drosophila* Genetics Reference Panel [115]. Despite the use of different mtDNAs and nuclear backgrounds, analyses of these genotypes have confirmed and extended the three lessons described above. The limited evidence for disruption of coadaptation, the larger effects of intraspecific mtDNA effects and the context-dependent nature of nuclear and environmental variation have all been repeated as major findings. Importantly, we have observed that the dietary environment influences phenotypic variation more so than either nuclear or mitochondrial variation (or their combination) [26] in highly canalized traits, e.g. development time.

Another repeatable result observed across studies is that the proportion of phenotypic variance explained by mtDNA genotype is much smaller than the variance explained by other main effects (e.g. nuclear genotype, environment, sex), when factorial designs are employed. This is true for both insects [26,53] and yeast [116]. Additional studies in *Drosophila* examining the influence of diet on mitochondrial effects reveal similar effects where factorial contrasts can be made [117,118]. An exception to this pattern comes from the *w501* epistasis described above, where a strong mtDNA effect can dominate the proportion of variance explained in limited 2 mtDNAs × 2 nDNAs designs [111], but the mtDNA effect of *w501* is diminished in larger mitonuclear genetic panels [105]. More recent RNA-seq analyses in *Drosophila* have revealed that the GxG effect is more pronounced on whole-organism phenotypes and less so on gene expression [27]. For example, the rank order of importance for phenotypic variation for gene expression is nDNA effects > mtDNA effects ≥ environment effects. By contrast, for development time, the rank order is environment effects > nDNA effects > mtDNA effects [26].

There are some other examples of strong mtDNA effects that show varying levels of repeatability. For example, Camus *et al*. [24] found a larger mtDNA effect on male longevity than on female longevity (*F*_male_ = 6.3, *p* = 0.0011; *F*_female_ = 0.71, *p* = 0.7), consistent with Mother's Curse. In a more recent paper using the same mtDNA strains on different diets, mtDNA effects were greater in females than males (proportion of variance for mtDNA effect in males on two diets were 0.0 and 0.14; the same values for females were 9.2 and 17.2% [119]). These data are not consistent with the Mother's Curse prediction, and the two studies reveal a strong context-dependent effect of mtDNA in different experiments. If one were to combine these studies in an effort to tabulate a general sex-specific effect size across both experiments, the effect sizes across mtDNAs would be greatly diminished. Again, the repeatable lesson from these studies is that the environment, the nuclear background or the sex of the fly can have much larger effect sizes than the mtDNA alone (figure 1*d* and table 1 in [26], and table 1 in [53]). While we are fond of the Mother's Curse idea and have published papers consistent with the phenomenon [3,7,23], our more recent data do not support the prediction. We find it troublesome that Mother's Curse gets treated as a phenomenon of mtDNA-driven selection when publications using common strains exist that provide both strong support and strong rejection of the primary prediction of Mother's Curse (larger effect sizes across mtDNAs in males than in females). An analogy comes from Haldane's Rule: this pattern has a few exceptions but it is a highly repeatable pattern seen in both male (XY) and female (ZW) heterogametic species. In short, Mother's Curse is a nice idea that has variable support, but it is a hypothesis, not a ‘rule’ illustrating genetic conflict.

**Figure 4.**
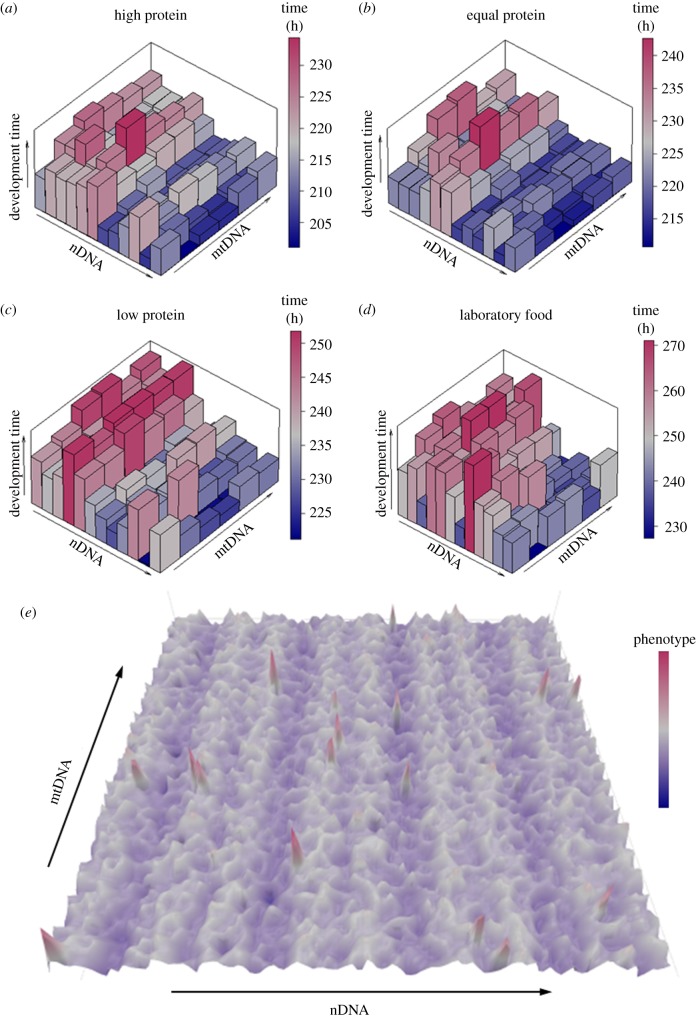
Empirical and simulated phenotypic landscapes. Dietary composition of altered protein (P): carbohydrate (C) geometry under isocaloric conditions alters development time in mitonuclear *Drosophila* strains (*a–d*) (data from [26]). Each figure contains a heat component corresponding with the magnitude of the phenotype (development time). The largest main effect is diet type (high P:C (*a*), equal P:C (*b*), low P:C (*c*) and laboratory food (*d*)) are shown. The second most important explanatory variable is the nuclear effect followed by mtDNA variation. Mitonuclear epistases are shown as mtDNA modification of nuclear effects. Mitonuclear epistasis can be both positive and negative. A simulated phenotypic landscape of mitonuclear variation (80 × nDNAs by 80 × mtDNAs = 6400 mitonuclear genotypes) in a single environment (*e*). The heat component corresponds with the magnitude of a phenotype. The major axis shows nuclear variation (nDNA), which consistently explains more of the variance in measured phenotypes than mtDNA (mtDNA). Mitonuclear epistases are shown as sharp peaks and troughs in the landscape. (Online version in colour.)

Understanding the complex three-way (gene × gene × environment (G×G×E)) interactions underpinning phenotypes will require an environment to be an explicit axis of variation in manipulative experiments. Environments can then be scrutinized individually (figure 2*b*) and together to identify the regions of gene–gene interaction (GGI) or protein–protein interaction (PPI) space that is consistently perturbed (figure 2*c*). Such experimental designs will allow identification of the regions of GGI or PPIs that are sensitive to mitonuclear variation. Lehner [73] provides the basis of an elegant ‘guilt-by-association’ framework to probe across-taxa mitonuclear variation with hierarchical phenotyping (e.g. using a combination of *in vitro*, *in vivo*, whole-organism studies, etc.). We have adopted this approach to scrutinize GxGxE in the *Drosophila* mitonuclear model (figures 2 and 4).

## 6. The keys to gene × gene × environment lie in anterograde and retrograde signalling

Complex higher-order genetic and environmental interactions are pervasive in *Drosophila* (figure 4) [26,27], and this has been demonstrated for genetic designs that do not explicitly model mtDNA variation [120,121]. In our gene expression studies, we find a large number of transcripts are differentially expressed across mitonuclear genotypes ([28]) and these are further influenced by abiotic environments (e.g. hypoxia [27] and diet [118]). These observations suggest that large sections of GGI and PPI networks are altered in experimental mitonuclear genotypes. The mechanisms underlying these effects must be governed by the myriad of signalling molecules that regulate anterograde (nucleus-to-mitochondria) and retrograde (mitochondria-to-nucleus) communication. Considerable recent advances have been made in identifying signalling mechanisms that transduce information about the intra- and extracellular environments to the nucleus. The mitochondrial unfolded protein response (UPR^mt^) [122–124] is a central component of the mitohormetic stress response to protein toxicity [125] and involves mitochondrial chaperones and proteases that help regulate whole-organism phenotypes through chromatin modifications [126–128]. Owing to its key role in homeostasis, the retrograde signalling cascade must be considered as an essential capacitor-like [129] phenotype to help integrate the genotype to phenotype landscape in variable environments [26].

## 7. Conflict and cooperation—negative and positive effects by another name

The terms ‘conflict’ and ‘cooperation’ represent two ends of a fitness continuum and we generally consider conflict as a deleterious outcome and cooperation as competent or high fitness state, with zero, negligible or positive phenotypic effects. However, how these phenotypes are generated and maintained in natural populations is poorly understood. For example, we can artificially introgress mtDNAs across species barriers with no obvious fitness consequences in *Drosophila* using balancer chromosomes and tricks of fly genetics. In this process, we are circumventing the role of hybrid breakdown and avoiding the first contact between mitonuclear alleles and mtDNA haplotypes that may have been co-adapted and isolated over speciation timescales. To what extent cryptic genetic variation is exposed during hybridization or in the generation of novel mitonuclear genotypes is unknown. In wild or outbred populations of a species, any novel mtDNA polymorphism and nuclear combination(s) will *always* be tested in heterozygous nuclear backgrounds in the F_1_ offspring. In almost all cases, the female in the cross carries mitonuclear genetic combinations that are tried and tested in a fitness sense. Hybridization will conceal fitness-related mitonuclear epistasis because the effects of recessive mitonuclear interactions are more likely to be experienced by the individual only in the next (F_2_) generation. This effect is clearly illustrated in between-population hybrids of the intertidal copepod *Tigriopus californicus*. Mitochondrial sequence divergence exceeding approximately 18% between populations can be successfully introgressed producing fertile F_1_ hybrids [130]. Hybrid breakdown is then revealed in the F_2_ generation and associated with an incompatibility between mtDNA-encoded *COX* and the nuclear-encoded cytochrome *c* (*Cytc*) gene [131]. Recent reciprocal F_2_ segregation assays of nuclear SNPs in alternative mtDNA backgrounds are an effective means of assessing the degree of conflict and cooperation spread across the nuclear chromosomes [132,133].

The macroevolutionary aspects of conflict and cooperation in mitochondrial evolution focus on competition or conflict among different mitochondria (or mtDNAs) within early proto eukaryotic cells. The assumption is that the competition would be deleterious to the ‘host’ nucleus and evolution would favour the elimination of this cytoplasmic polymorphism. There is some experimental evidence that is consistent with this deleterious-heteroplasmy scenario in a mouse model [134]. A more recent study of a large number of mother–offspring pairs revealed the nuclear genetic background can exert a force of selection that keeps deleterious heteroplasmic mutations at a minimum [135]. While these fully eukaryotic model systems are distinct from the early cytoplasms that shaped eukaryotes, these kinds of analyses may shed light on how conflict and cooperation were resolved in the early stages of eukaryotic evolution.

## 8. Concluding remarks

Chetverikov's vision of a genotypic environment in 1926 [56] forecasted the basis of our current understanding of mitonuclear epistatic interactions. Mitonuclear interactions are pervasive and have pleiotropic effects across numerous phenotypes. Understanding the genetic and physical arenas of conflict and cooperation in this co-evolved unit will delineate the first-order and higher-order genetic and environmental effects on fitness and disease. Here we have outlined some approaches that could help elucidate the phenotypic and fitness landscapes and their relation to genetic variation. Mitochondria play a special role in dissecting this genotype–phenotype map. It is our opinion that focusing on the pervasive effects of GxG and GxE interactions and the critical importance of understanding marginal effect sizes for mtDNA across multiple backgrounds should drive the future of research in this field (figure 1).

An important caveat for this view of mtDNA effect size is the strength of the population structure. In species with a strong population structure (high *F*_ST_ for both nuclear and mtDNA), each mtDNA haplotype might experience only a few alternative nuclear genotypes with which to interact. Likewise, a strong population structure might limit the range of external environmental factors that any mitonuclear genotype would experience (although strong diurnal and seasonal abiotic selection could still exist). In species with strong population structure, we might see stronger opportunities for mitonuclear coadaptation, and its breakdown by mitonuclear ‘transplant’ experiments. In species with little population structure and high allelic variation, mtDNA and nuclear variants would experience multiple different ‘backgrounds’, and the opportunity for tight coadaptation may be limited; however, the opportunity for epistasis and sex-specific mitonuclear effects could be greatly enhanced. This population structure-mitonuclear coadaptation hypothesis could help explain why the breakdown of mitonuclear coadaptation is so evident in *Tigriopus*, which has highly structured inbred populations, while this is not seen in large outbred species such as *Drosophila*.

It is easy to construct mitochondrial genotypes that might generate phenotypic effects that can appear supportive of broad evolutionary trends in single publications, but interpreting these effects as proof that mitochondria have driven the origin of sex, speciation, pervasive genomic conflict, Mother's Curse or other interesting ideas needs to be based on a broad set of studies that fully examine context-specific effects. Clearly, there is much still to learn.

## Data accessibility

This article does not contain any additional data.
